# Levels of Alpha-Toxin Correlate with Distinct Phenotypic Response Profiles of Blood Mononuclear Cells and with agr Background of Community-Associated *Staphylococcus aureus* Isolates

**DOI:** 10.1371/journal.pone.0106107

**Published:** 2014-08-28

**Authors:** Srikanth Mairpady Shambat, Axana Haggar, Francois Vandenesch, Gerard Lina, Willem J. B. van Wamel, Gayathri Arakere, Mattias Svensson, Anna Norrby-Teglund

**Affiliations:** 1 Karolinska Institutet, Centre for Infectious Medicine, Stockholm, Sweden; 2 CIRI, International Center for Infectiology Research, LabEx Ecofect, Université Lyon 1, Inserm U1111, Ecole Normale Supérieure de Lyon, CNRS UMR5308, Centre National de Référence des Staphylocoques, Hospices civils de Lyon, Lyon, France; 3 Department of Medical Microbiology and Infectious Diseases, Erasmus Medical Centre, Rotterdam, Netherlands; 4 Society for Innovation and Development, Indian Institute of Science Campus, Bangalore, India; University of Edinburgh, United Kingdom

## Abstract

Epidemiological studies of *Staphylococcus aureus* have shown a relation between certain clones and the presence of specific virulence genes, but how this translates into virulence-associated functional responses is not fully elucidated. Here we addressed this issue by analyses of community-acquired *S. aureus* strains characterized with respect to antibiotic resistance, ST types, *agr* types, and virulence gene profiles. Supernatants containing exotoxins were prepared from overnight bacterial cultures, and tested in proliferation assays using human peripheral blood mononuclear cells (PBMC). The strains displayed stable phenotypic response profiles, defined by either a proliferative or cytotoxic response. Although, virtually all strains elicited superantigen-mediated proliferative responses, the strains with a cytotoxic profile induced proliferation only in cultures with the most diluted supernatants. This indicated that the superantigen-response was masked by a cytotoxic effect which was also confirmed by flow cytometry analysis. The cytotoxic supernatants contained significantly higher levels of α-toxin than did the proliferative supernatants. Addition of α-toxin to supernatants characterized as proliferative switched the response into cytotoxic profiles. In contrast, no effect of Panton Valentine Leukocidin, δ-toxin or phenol soluble modulin α-3 was noted in the proliferative assay. Furthermore, a significant association between *agr* type and phenotypic profile was found, where *agr*II and *agr*III strains had predominantly a proliferative profile whereas *agr*I and IV strains had a predominantly cytotoxic profile. The differential response profiles associated with specific *S. aureus* strains with varying toxin production could possibly have an impact on disease manifestations, and as such may reflect specific pathotypes.

## Introduction

*Staphylococcus aureus* (*S. aureus*) is a significant cause of human infections and an emerging health problem globally due to its increasing resistance to beta-lactams (methicillin-resistant *S. aureus*, MRSA). A special cause of concern is the rise in community-acquired (CA) *S. aureus* strains, and particularly concerning are reports of specific CA-MRSA clones associated with highly aggressive infections, including necrotizing fasciitis and pneumonia in otherwise healthy individuals [1]–[3]. Although there are large geographical differences, epidemiological studies have shown that more than 20 distinct CA-MRSA lineages are present globally [4].

The pathogenesis of invasive staphylococcal infections involves a variety of virulence factors. In severe invasive infections, several exotoxins have been implicated in disease pathogenesis, such as superantigens [5]–[7], as well as the cytotoxins Panton Valentine Leukocidin (PVL) [8]–[10], alpha-toxin (α-toxin) [8], [10], and phenol soluble modulins (PSMs) [10], [11]. There are to date 24 distinct superantigens identified in *S. aureus*, including the well characterized TSST-1 and the staphylococcal enterotoxins (SE) A–C. Superantigens have been attributed a central role in mediating the systemic toxicity and shock by virtue of their ability to induce hyper-inflammatory responses [6], [7], and they have also been implicated in severe pulmonary infections [5]. Cytotoxins have been associated with cell and tissue injury as well as inflammation in severe invasive infections, including necrotising infections of the skin and lung [10]. Several of the toxins are encoded by mobile genetic elements resulting in great diversity in toxin gene content among *S. aureus* strains [12], [13] Many of these virulence factors are regulated and controlled by a global regulator system called the accessory gene regulator (agr) system encoded by the *agr* locus [14]–[17]. The *agr* locus has diverged among different *S. aureus* strains with polymorphism in *agrBDC* region, resulting in four major allelic types of *agr*, i.e. *agr*I–IV [15], [17], [18]. A recent study demonstrated the impact of allelic variation on *agr* induction dynamics, which translated into significant differences in expression of several virulence factors [19].

*S. aureus* infected patients have been shown to develop antibodies against both superantigens and cytotoxins [20]–[24]; thus demonstrating that patients are exposed to a combination of exotoxins secreted by the strains during infection. Exactly how these different combinations of toxins affect virulence and disease outcome is, as of yet, not fully elucidated. One example of interactions between superantigens and cytotoxins was reported by Broshnan et al [25] who showed that cytolysins promoted increased penetrance of superantigens in mucosa. Here we set out to explore how diversity in exotoxin profiles among CA *S. aureus* strains translates into virulence-associated functional responses. To this end, we determined the effect on peripheral blood mononuclear cells (PBMC) elicited by CA *S. aureus* strains of different sequence types (ST) with distinct *agr* allelic types and toxin gene profiles. Our data revealed that the strains induced differential responses in PBMC, characterized by either cytotoxic or proliferative responses, which were linked to specific *agr* types and levels of α-toxin expression.

## Materials and Methods

### Ethics Statement

This study includes blood samples from buffy coats of blood provided by the blood bank at the Karolinska University Hospital. The buffy coats were provided anonymously; hence informed consent was not required. The ethical research committee at Huddinge University Hospital (Forskningskommitte Syd) approved the study.

### Clinical isolates

Strains (n = 38) were selected from a heterogeneous cohort of CA *S. aureus* representing a diverse collection of isolates with different ST and toxin profiles collected from colonized individuals (nasal swabs) or patients with varying *S. aureus* infections in India (Table 1). The isolate collection has previously been characterized with respect to antibiotic resistance profile, molecular typing including ST, *agr*-types, and toxin profile as determined by either PCR for specific genes or by a DNA microarray covering 185 *S. aureus* genes [26]. The study also included a confirmatory cohort of 31 isolates collected from patients with CA *S. aureus* pneumonia included in a prospective cohort study in France [9] or from cases referred to the French national reference laboratory for staphylococci.

**Table 1:** Characteristics of *S. aureus* strains with respect to antibiotic resistance, ST and agr types, toxin genotype and production i*n* *vitro.*

Strains	ClinicalDiagnosis	MSSA/MRSA	*agr* type	SPA type	CC/ST type	*pvl*	*luk*D/E	*se/tsst-1/egc*	*Etd/edinB*	ResponseProfile#	Luminex*
***Colonizing*** ***strains***											
Sa559		MSSA	I	t005	ST22/CC22	+	ND	−/−/+	−	C (96.6)	α-toxin+++
Sa115		MRSA-IV	I	t852	ST22/CC22	+	ND	−/−/+	−	P (12.3)	α-toxin +
Sa165		MRSA-IV	I	t852	ST22/CC22	+	ND	−/−/+	−	C (95.7)	α-toxin +++
Sa95		MSSA	I	t3840	ST672	−	+	−/−/+	−	C (98.0)	α-toxin +++
Sa134		MRSA-V	I	t064	ST1208/CC8	+	+	*se*-A, B, K, Q/−/−	−	P (76.5)	α-toxin ++/SEB++
Sa180		MSSA	I	t4285	ST6	−	ND	*se*-L/*tsst-1*/−	−	C (97.5)	α-toxin +++/TSST+++
Sa168		MSSA	I	t937	ST291	−	−/+	−/−/−	+	C (96.7)	α-toxin +++
Sa337		MSSA	I	t3096	ST291	−	ND	−/−/−	+	C (96.1)	α-toxin +++
Sa18		MRSA-V	II	t657	ST772/CC1	+	−	*se*-A, C, L/−/+	−	P (27.0)	α-toxin ++
Sa289		MSSA	II	t1839	ST772/CC1	+	−	*se*-A/−/+	−	P (28.5)	α-toxin ++
Sa1437		MSSA	II	t345	ST772/CC1	+	ND	*se*-A, C, L/*tsst*-1/+	−	P (23.7)	α-toxin +
Sa1446		MRSA-V	II	t657	ST772/CC1	+	ND	*se* - A, E/−/+	−	P (25.4)	α-toxin +
Sa233		MRSA-V	II	t657	ST772/CC1	+	ND	*se*-A, C, E, L/−/+	−	P (30.0)	α-toxin ++
Sa159		MSSA	II	t774	ST199/CC15	−	+	−/−/−	−	Ambigious	α-toxin +
Sa160		MSSA	II	t774	ST199/CC15	−	+	−/−/−	−	C (69.6)	α-toxin +
Sa32		MRSA-IV	III	t021	CC30	+	−	−/−/+	−	P (21.2)	α-toxin +/−
Sa368		MSSA	IV	t1999	CC121	+	ND	−/−/+	−	C (93.7)	α-toxin +++
Sa14		MSSA	IV	t3204	CC121	+	−/+	*se*-B/−/+	−	C (95.0)	α-toxin +++/SEB+++
***Disease strains***											
Sa37	SSTI	MRSA-IV	I	t852	ST22/CC22	+	−	−/−/+	−	P (12.8)	α-toxin +
Sa08	SSTI	MRSA-IV	I	t852	ST22/CC22	+	−	−/−/+	−	C (98.0)	α-toxin +++
Sa113	Brain abscess	MRSA-IV	I	t852	ST22/CC22	+	−	−/−/+	−	C (95.0)	α-toxin +++
Sa114	Cerebral abscess	MRSA-IV	I	t852	ST22/CC22	+	−	−/−/+	−	C (95.8)	α-toxin +++
SaN08	Meningitis	MRSA-IV	I	t852	ST22/CC22	+	−	−/−/+	−	C (96.5)	α-toxin +++
Sa1	Invasive infection	MRSA-IV	I	t1309	ST672	−	+	−/−/+	−	C (94.6)	α-toxin +++
Sa754	Invasive infection	MRSA-IV	I	t852	ST22/CC22	+	−	−/−/+	−	C/P (87.3)	α-toxin +++
Sa755	Invasive infection	MSSA	I	ND	ST22/CC22	+	−	−/−/+	−	P (3.31)	α**-**toxin ++
Sa118	Pyomyositis	MRSA-V	II	t657	ST772/CC1	+	−	*se*-A, C, L/−/+	−	P (30.7)	α-toxin ++
Sa3957	Breast abscess	MRSA-V	II	t1387	ST772/CC1	+	ND	*se*-A, E/−/+	−	P (21.5)	α-toxin +/−
Sa3989	Pneumonia	MRSA-V	II	t3596	ST772/CC1	+	ND	*se*-A, E/−/+	−	P (24.8)	α-toxin ++
Sa120/1	Cerebral abscess (pus)	MRSA-V	II	t657	ST772/CC1	+	ND	ND	ND	P (24.7)	α-toxin ++
Sa2332	Pleural Empyema	MSSA	III	t021	CC30	+	−	−/−/+	−	P (36.2)	α-toxin +/−
SaP1	Suture induced infiltrate	MSSA	III	t127	ST1	−	ND	*se* **-**D, E/−/−	−	P (80.5)	ND
SaP3	Keratitis	MSSA	III	t8078	ST1	−	ND	*se*-D/−/−	−	P (27.1)	ND
SaP6	Keratitis	MSSA	III	t127	ST1	−	ND	*se*-D, E/−/−	−	P (60.1)	ND
SaP7	Orbital abscess	MRSA-V	III	t2526	ST88	+	ND	*se*-D/−/+	−	C (97.3)	ND
Sa753	Necrotizing pneumonia	MSSA	IV	t159	ST121	+	+	−/−/+	−	C (98.3)	α-toxin +++
Sa796	Necrotizing pneumonia	MSSA	IV	t159	ST121	+	+	−/−/+	−	C (97.4)	α-toxin +++
Sa1059	Invasive inf.	MSSA	IV	t159	ST121	+	+	−/−/+	−	C (98.5)	α-toxin +++

MRSA, methicillin-resistant S. aureus; MSSA, methicillin-susceptible S. aureus; ND, not determined; *se*, staphylococcal enterotoxin;P, proliferative; C, cytotoxic; SSTI, skin and soft tissue infection.

# The response profile is determined by the pattern of proliferative responses elicited by different dilutions of bacterial supernatants. A proliferative profile was denoted if all dilutions elicited a proliferative response. A cytotoxic pattern was denoted if a) proliferation was only noted at the highest dilution of the supernatants, and b) the supernatants (1∶50 dilution) resulted in inhibition of PHA-induced responses in co-stimulation experiments. Cells stimulated with supernatants (1∶50 dilution) were also analyzed by flow cytometry and the value in parenthesis shows % cells stained positive for dead cell marker.

*Analyses of selected exotoxins including α-toxin (alpha-toxin), SEB (staphylococcal enterotoxin B) and TSST-1 (toxic shock syndrome toxin 1) are assessed in overnight bacterial culture supernatants by luminex. Only positive results are indicated and based on the dilution series response a semi quantitative measure is given as +/−, +, ++, +++.

A clinical USA300 strain (LUG2012) from a patient from South-Ouest of France and its isogenic mutants deficient for either α-toxin (LUG2209) or PVL (LUG2040) were also included to confirm toxin-mediated effects. The deletion mutants were obtained by using pMAD [27] carrying the replacement cassette that was electroporated to RN4220 recipient strain and then to LUG2012. Growth at non-permissive temperature (44°C) was followed by several subcultures at 30°C and 37°C to favor double crossing over as previously described [28]. Validation of deletion of the *hla* (LUG2209) or *luk*SF-PV (LUG2040) genes were done by PCR and the production of α-toxin and PVL were determined with previously described toxin-specific ELISA (see below).

### Preparation of bacterial culture supernatants

The strains were cultured overnight at 37°C in 25 ml casein hydrolysate and yeast extract (CCY) medium. Cell-free supernatants were prepared through centrifugation at 3350 g followed by filter sterilization.

### Toxin determination

Bacterial supernatants were assessed for the presence of selected exotoxins, including α-toxin, staphylococcal enterotoxin B (SEB), and toxic shock syndrome toxin 1 (TSST-1), using a multiplex competition immunoassay based on Luminex technology [29], and the amounts of α-toxin and PVL in the bacterial supernatants were determined by specific ELISA as previously described [30], [31] using specific antibodies kindly provided, respectively, by GSK Biologicals Inc. (USA) and bioMerieux R&D Immunodiagnostic (France).

### PBMC proliferation assay

Human PBMC were isolated from healthy donors using Lymphoprep density centrifugation. The cells were cultured in RPMI-1640 medium supplemented with 10% FCS, 10 mM of L-glutamine, Penicillin (100 U/ml)/Streptomycin (100µg/ml) and 25 mmol/L HEPES (all from Thermo Scientific HyClone, USA). PBMC were seeded at 2×10^5^ cells/well and stimulated at 37°C with serial dilutions of bacterial supernatants. After 72 hours, the cells were pulsed for 6 hours with 1 µCi/well of 3H-thymidine (Perkin-Elmer) after which 3H-uptake was measured in a beta-scintillation counter. Phytohemagglutinin-L (PHA) (1 µg/ml) (Sigma-Aldrich, St. Louis, USA) was used as a positive control for polyclonal T cell activation. The cytotoxic/inhibitory effect was tested by the addition of bacterial supernatants in combination with PHA in the proliferation assay. The bacterial culture medium CCY was included as a negative control, and was found to have negligible effect on proliferation (mean CPM 3088, 1785, 1719 for 1∶50; 1∶100 and 1∶1000 dilution, resp.) and no inhibitory effect on PHA-induced proliferation. PBMC were also stimulated with bacterial supernatants or PHA in combination with purified α-toxin (Sigma-Aldrich, St. Louis, USA), recombinant PVL, recombinant δ-toxin and purified PSM-α3 (all from IBT Bioservices, Gaithersburg, USA) and proliferation assessed.

### IVIG inhibition assay

PBMC were stimulated with bacterial supernatants, α-toxin or PHA in the presence or absence of different concentrations of IVIG (Gammagard S/D, Baxter). Proliferative responses were determined after 72 hours as described above.

### Flow cytometry analysis

Stimulated PBMC were washed and incubated for 30 minutes on ice with directly conjugated antibodies (CD3: SK7, R&D Systems; CD45: T29/33, BD Biosciences; HLA-DR: TU36, Life technologies) in combination with a dead cell marker (Live/Dead Fixable near IR; Molecular Probes). Analyses were done using a Beckton Dickinson LSRII SORP flow cytometer and FlowJo 9.5.3.

### Statistical evaluation

Data were analyzed by GraphPad Prism version 4.0 for Windows (GraphPad software). Two-sided Mann-Whitney *U* test or Fisher’s exact test were used for comparison between two groups. Comparisons between multiple groups were done using ANOVA and Dunn's multiple comparison test. Differences were considered significant when *p*<0.05.

## Results

### Distinct functional phenotypic profiles of clinical *S. aureus* strains

A proliferation assay was employed in which PBMC were exposed to bacterial supernatants prepared from CA *S. aureus* strains. The strain collection included CA MRSA and MSSA strains of varying ST types, *agr* types and toxin gene profile isolated from patients or colonized individuals (Table 1). All strains, except two (strains Sa159 and Sa160), harbored superantigen encoding genes (Table 1). To confirm that the genes were expressed during *in vitro* culture, the bacterial supernatants were analyzed for toxin content using a customized luminex assay. Due to technical limitations only a selected set of exotoxins, i.e. α-toxin, SEB and TSST-1, were included for which the protein detection concurred with the toxin gene profile of respective strain (Table 1).

**Figure 1:**
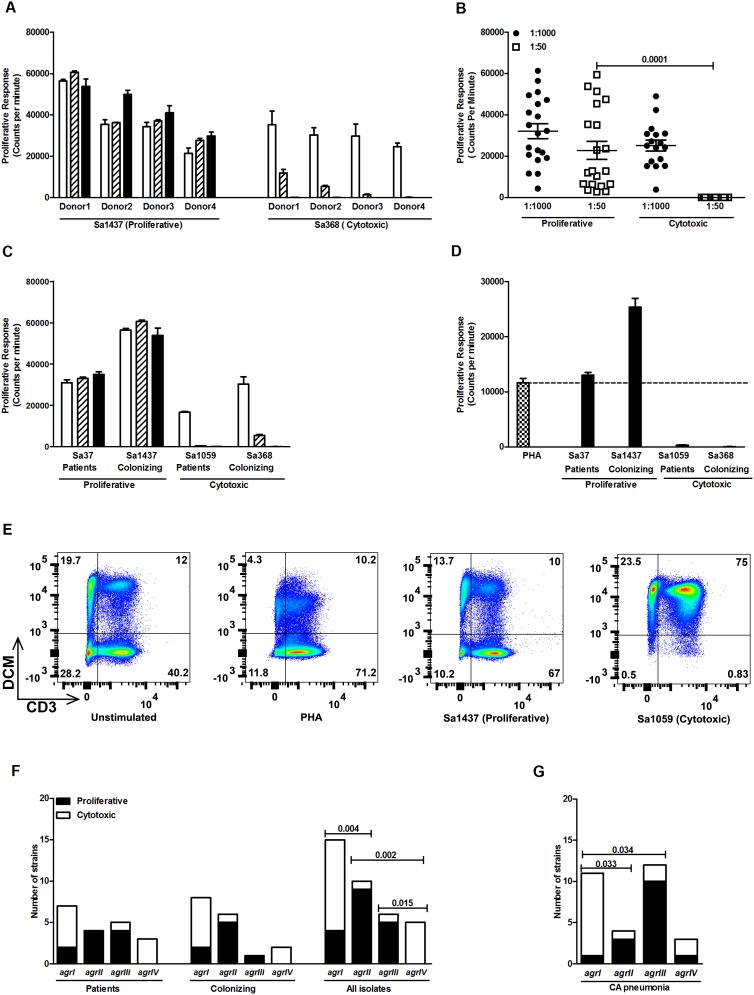
Proliferative or cytotoxic responses elicited by *S. aureus* strains. Human peripheral blood mononuclear cells (PBMC) isolated from healthy donors were stimulated with dilution series (1∶1000 (open), 1∶100 (stripped) and 1∶50 (filled) bars) of bacterial supernatants prepared from overnight cultures of *S. aureus* strains. Proliferative responses were determined by 3H-thymidine uptake and are presented as mean counts per minute ± SD. The supernatants induced either a proliferative or cytotoxic response profile. A cytotoxic profile was assigned when strains did not elicit a proliferative response in the more concentrated supernatants but only in the most diluted samples. A) Shows the response of a representative proliferative and cytotoxic supernatant in separate experiments using cells from four healthy donors. B) Scatter plot of proliferative responses induced by bacterial supernatants with a proliferative or a cytotoxic profile. Mean values of four different experiments are shown for dilutions 1∶1000 (filled symbols) and 1∶50 (open symbols). C) Bacterial supernatants with proliferative or cytotoxic profiles were found among both patients (n = 20) and colonized individuals (n = 17). The figure shows proliferative responses (mean ± SD) of one representative, out of five, experiments using cells from different donors stimulated with dilution series (1∶1000 (open), 1∶100 (stripped) and 1∶50 (filled) bars) of bacterial supernatants. D) To confirm a cytotoxic effect, proliferative responses were assessed in cells stimulated with PHA alone (the dashed line shows the average PHA response) or in combination with supernatants (proliferative and cytotoxic; dilution 1∶50 (filled) bars). The figure shows one representative of three experiments using cells from different donors. E) Flow cytometry analysis on PBMC stimulated with PHA and bacterial supernatants (1∶50 dilution). Total PBMC were gated based on CD45 expression and CD45 positive cells were further analysed for dead cell marker (DCM, Y axis) and CD3 (X axis) positivity. The figure shows one representative of three individual experiments using cells from different donors. F) Relation between *agr* types and proliferative (black bars) or cytotoxic (white bars) community *S. aureus* strains collected from colonizing individuals (n = 17) or patients with infections (n = 20) in India. G) Relation between *agr* type and proliferative or cytotoxic profiles elicited by community-acquired (CA) pneumonia patients (n = 31). Statistical significant differences were determined by use of the two-sided Mann Whitney test and Fisher's exact test with two sided p value and p values are indicated in the figure.

PBMC from four different donors were stimulated with serial dilutions of the bacterial supernatants and proliferative responses assessed (Figure 1A). This assay is commonly used to functionally assess superantigen-mediated proliferation, but the response will be influenced by the presence of cytotoxins targeting PBMC. Accordingly, supernatants prepared from the different *S. aureus* strains induced starkly different (p<0.0001) and highly reproducible response profiles (Figures 1A, 1B). While some strains induced consistently high proliferative responses at all dilutions (1∶50, 1∶100 and 1∶1000) of the bacterial supernatants (denoted as a proliferative profile) (Figures 1A, 1B), other strains induced proliferation only at the highest dilution (1∶1000) of bacterial supernatants (Figures 1A, 1B). Thus, demonstrating that the supernatants contain superantigens that trigger proliferation, but this activity is masked in more concentrated supernatants from certain strains, potentially through toxin-mediated cytotoxicity.

To investigate this further, proliferation was assessed following stimulations of PBMC with the polyclonal T cell activator PHA in combination with supernatants displaying a proliferative or cytotoxic profile (Figure 1C). Using the 1∶50 dilutions of bacterial supernatants, the PHA-response was completely abolished by the cytotoxic supernatants, whereas augmented by the proliferative supernatants (Figure 1D). To directly assess whether bacterial supernatants were cytotoxic, flow cytometry analysis was applied on cells stimulated with supernatants and subsequently stained with a dead cell marker in combination with antibodies directed towards defined cell markers. PBMC stimulated with a proliferative supernatant showed a relative expansion of the T cell population similar to that seen for PHA (Figure 1E). Notably, stimulation with a cytotoxic supernatant resulted in extensive cell death as almost all cells stained positive for the dead cell marker (Figure 1E). As shown in Table 1, cell death was significantly more pronounced among cells exposed to cytotoxic, as compared to proliferative, supernatants (mean % of cells staining positive for dead cell marker: 96.3% and 33.2% for cytotoxic and proliferative supernatants, resp.; p<0.001).

The two functional response profiles were found in both colonizing as well as patient isolates and there was no significant association between functional profile and ST-type/clonal complex or MRSA/MSSA type (Table 1). In contrast, an association with *agr* type was evident, and *agr* II and *agr* III strains had predominantly a proliferative profile whereas *agr* I and IV strains were cytotoxic (p<0.015) (Figure 1F). As these analyses were conducted on a highly heterogeneous strain cohort, we expanded the analyses to include a more homogenous strain cohort collected from patients with CA *S. aureus* pneumonia. Also in this cohort, a similar association with *agr* I and IV being significantly more cytotoxic than *agr* II or III was noted (p<0.034) (Figure 1G).

### High α-toxin expression is linked to the cytotoxic profiles

**Figure 2:**
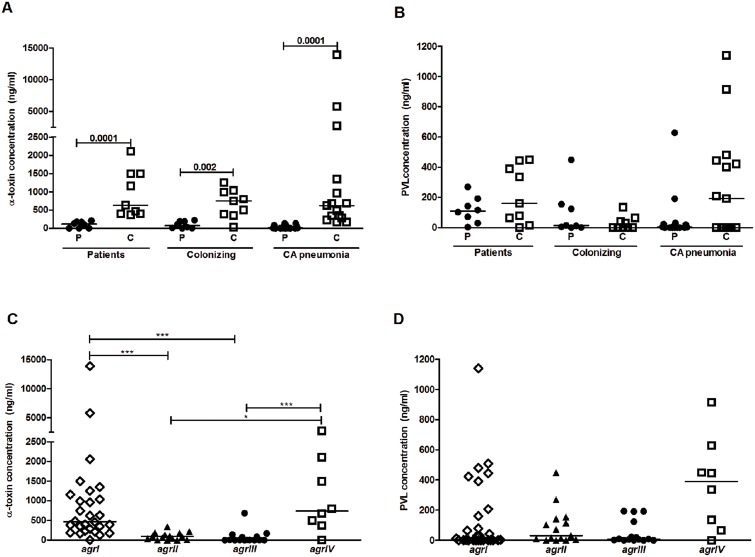
Levels of alpha-toxin expression correlate with cytotoxic profile and *agr* types. Amounts of alpha-toxin (α-toxin) (A) and Panton Valentine Leukocidin (PVL) (B) were determined by ELISA in the *S. aureus* bacterial supernatants (1∶50 dilutions) having either a cytotoxic (C; open symbols) or a proliferative (P; filled symbols) response profile. Supernatants prepared from isolates collected from colonized individuals (n = 17), patients (n = 17), or a confirmatory cohort of CA pneumonia (n = 31) are shown. Association between amount of α-toxin (C) and PVL (D) in the *S. aureus* bacterial supernatants (1∶50 dilutions; n = 65) with their respective *agr* types (*agr* I and IV, open symbol; *agr* II and III, filled symbol). Statistical significant differences were determined by use of the two-sided Mann Whitney test or with ANOVA and Dunn's multiple comparison test for comparisons of multiple groups and p values are indicated in the figure.

Quantitation of the cytotoxins α-toxin and PVL revealed that the cytotoxic supernatants had significantly higher levels of α-toxin than the proliferative, regardless of whether the strains were collected from patients, colonized individuals, or CA pneumonia (p<0.002) (Figure 2A). In contrast, there was no correlation between the response profile and PVL expression (Figure 2B) which is in line with the reported lack of susceptibility of PBMC to PVL [32]. In agreement with the data in Figure 1F demonstrating that different *agr* types are linked to either proliferative or cytotoxic profiles, significantly higher amounts of α-toxin were found in strains belonging to the cytotoxic *agr* type I and IV, as compared to the proliferative *agr* type II and III strains (p<0.05) (Figure 2C). In this context, no association between PVL levels and specific *agr* types was seen (Figure 2D). Also, expression data on the *psm*-α gene revealed no difference between strains eliciting either response profile (data not shown).

**Figure 3:**
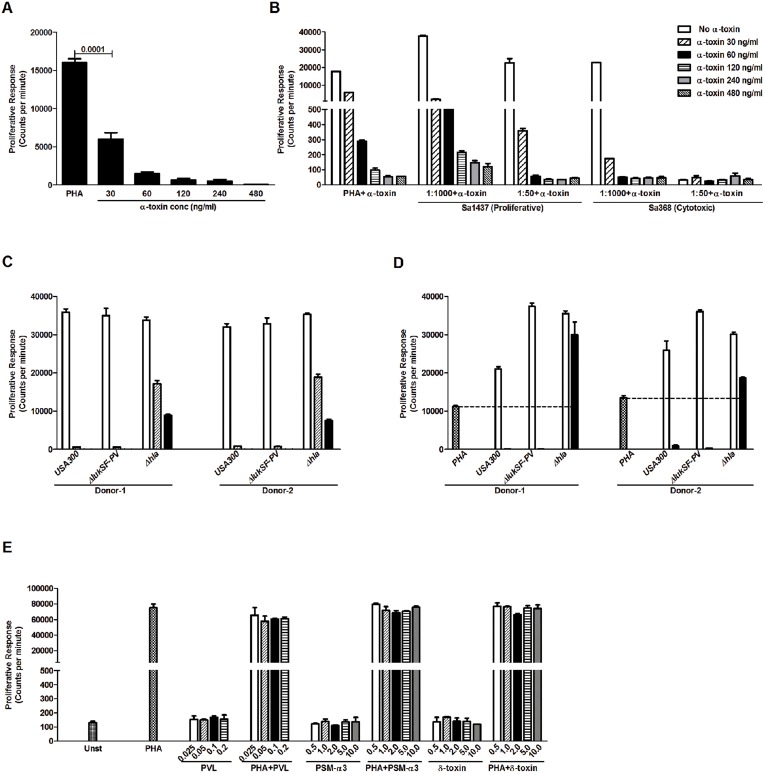
Alpha-toxin mediates cytotoxicity in PBMC. A) Proliferation assay using human PBMC stimulated with PHA alone or in the presence of different concentrations of α-toxin. Proliferative responses were determined by 3H-thymidine uptake and are presented as mean counts per minute ± SD. Mean values of four different experiments from different donors are shown. B) Inhibition of PHA-induced and bacterial supernatant-induced proliferation of PBMCs by addition of increasing concentrations of α-toxin (60 to 480 ng/ml). The figure shows one representative of two experiments using cells from different donors. C) Proliferation assay using cells from two donors stimulated with different dilutions (1∶1000 (open), 1∶100 (stripped) and 1∶50 (filled) bars) of supernatants prepared from USA300 (LUG2012), and its isogenic mutants of PVL (Δ*lukSF-PV*) and α-toxin (Δ*hla*). D) Proliferative responses assessed in cells stimulated with PHA alone or in combination with indicated supernatants at dilutions 1∶1000 (open) and 1∶50 (filled) bars. The figure shows experiments using cells from two donors. E) PBMC were stimulated with increasing concentrations of PVL, PSM α3 and δ-toxin at indicated concentrations (µg/ml) alone and in combination with PHA. Statistical significant differences were determined by Fisher's exact test with two sided p value and p value is indicated in the figure.

These data implied that α-toxin is a main mediator of the noted cell death in PBMC. To further test this, PBMC were stimulated with PHA in combination with purified α-toxin revealing a clear dose response pattern where increasing amounts of α-toxin resulted in reduced proliferation (Figure 3A). Furthermore, supplementing a proliferative (Sa1437) or a cytotoxic (Sa368) *S. aureus* supernatant with purified α-toxin, resulted in a switch to a cytotoxic response both at the 1∶1000 and 1∶50 dilutions for the proliferative strain and an increased cytotoxic response at the 1∶1000 dilution for the cytotoxic strain (Figure 3B). Further support for an α-toxin-mediated effect was provided by using a strain of the USA300 lineage (LUG2012), which belongs to the cytotoxic *agr* type I and produces high levels of α-toxin (α-toxin 83 µg/ml, PVL 14 µg/ml), and its isogenic mutants for PVL (α-toxin 54 µg/ml, PVL 0 µg/ml) and α-toxin (α-toxin 0 µg/ml, PVL 11 µg/ml). The USA300 and the PVL-deficient mutant both showed a cytotoxic profile, whereas the α-toxin mutant showed a proliferative response profile (Figure 3C). Also when PBMC were stimulated with PHA in combination with these supernatants a reduction of PHA-induced responses was noted in the presence of supernatants from USA300 and the PVL-deficient mutant (Figure 3D). In contrast, the α-toxin mutant supernatant resulted in an augmentation of the PHA-induced response (Figure 3D). Flow cytometry analysis further confirmed a potent cytotoxic effect of USA300 and the PVL-deficient mutant supernatants (97.2% and 85.8% dead cell marker positivity, resp.), whereas the α-toxin mutant supernatant had limited cytotoxicity (20.4% dead cell marker positivity). It should be noted that although the supernatant of the α-toxin mutant elicited a proliferative response at all dilutions tested, the proliferative response increased with more diluted supernatants (Figure 3C); thus, indicating that there are inhibitory factors, other than α-toxin, present in the USA300 supernatant. Other cytotoxins tested, including purified PVL, PSM-α3 and δ-toxin alone or in combination with PHA did not elicited any cytotoxic or proliferative responses in PBMC (Figure 3E).

### IVIG inhibition of toxin mediated responses in PBMC

**Figure 4:**
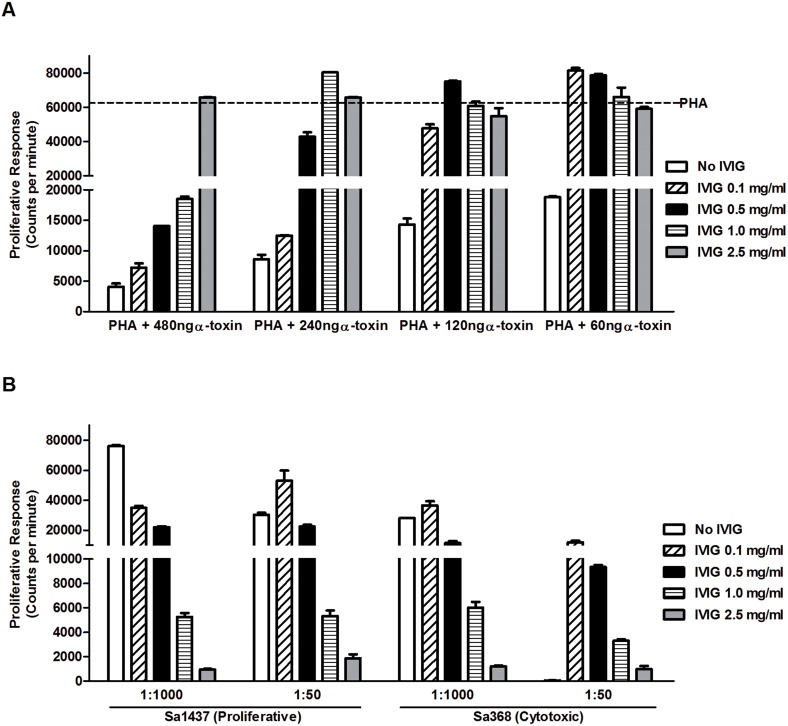
Inhibition of α-toxin and staphylococcal supernatant mediated cytotoxicity and proliferation of PBMC by IVIG. A) Neutralization of α-toxin induced cytotoxicity of PBMC by different concentrations of IVIG. PBMC were stimulated with PHA with increasing concentrations of α-toxin (60 to 480 ng/ml) in the presence or absence of IVIG (0.1 to 2.5 mg/ml, as indicated). The dashed line indicates the mean PHA-induced proliferative response. B) Proliferative responses induced by bacterial supernatants from both proliferative and cytotoxic strains (dilutions 1∶1000 and 1∶50) in the presence or absence of different concentrations of IVIG (0.1 to 2.5 mg/ml, as indicated). 3H-thymidine uptake after 72 hours of culture is presented as mean counts per minute ± SD. The figure shows one representative of two experiments using cells from different donors.

Studies have demonstrated the presence of antibodies against defined *S. aureus* virulence factors, including α-toxin, PVL and superantigens, in intravenous polyclonal immunoglobulin (IVIG) preparations [33]–[37]. Here we tested whether IVIG could inhibit the toxin-mediated functional response profiles, in particular the cytotoxic effect associated with high α-toxin levels. To this end, PBMC were stimulated with PHA and different concentrations of purified α-toxin in the presence or absence of IVIG. We found a clear dose response of IVIG-mediated inhibition of α-toxin cytotoxicity, evident by increased proliferative responses (Figure 4A). At the lowest concentration of α-toxin (60 ng/ml), a significant inhibition was achieved with 0.1 mg/ml IVIG (p = 0.05) while at the highest concentration of 480 ng/ml of α-toxin significant neutralization was achieved only at concentrations >1.0 mg/ml of IVIG (p = 0.034) (Figure 4A). Similarly we found that IVIG was able to inhibit both the proliferative as well as the cytotoxic responses elicited by superantigens and cytotoxins present in *S. aureus* culture supernatants (Figure 4B).

## Discussion

In this study we demonstrate that CA *S. aureus* strains with distinct toxin profiles exhibit stable robust phenotypic profiles evident by their ability to elicit either a proliferative or cytotoxic response profile in human PBMC. All experiments were done using bacterial supernatants containing superantigens and cytotoxins secreted by the strains. The data shows that the vast majority of strains elicit superantigenic activity as demonstrated by the induction of proliferative responses in PBMC. However, this response was masked in cytotoxic strains due inhibitory/cytotoxic factors present in the supernatants. This illustrates the relevance of using such a mixture of secreted factors from clinical isolates, which allows simultaneous analyses of the combined activity of toxins and which more closely reflects what the patients are exposed to. The PBMC assay provided an efficient tool to assess both superantigenic and cytotoxic effects; thereby allowing for identification of distinct phenotypic response profiles among CA S. *aureus* isolates. However, it should be noted that this assay involves only PBMC and hence, excludes effects of pore-forming toxins, such as PVL, LukDE and LukAB, that targets cell populations which are either missing or represent a minor subset in PBMCs [32], [38], [39]. Therefore, future studies should include also other clinically relevant cells, such as neutrophils, that are highly susceptible to the above mentioned cytotoxins.

Our findings demonstrated a striking association between high α-toxin levels and a cytotoxic phenotypic profile of the *S. aureus* strains. Supernatants that displayed a cytotoxic profile had in average 20-fold higher α-toxin levels than the proliferative supernatants (p<0.0001). Furthermore addition of purified α-toxin to either bacterial supernatants or to PHA resulted in a dose-dependent transition from proliferative to cytotoxic response profiles. Also, a USA300 α-toxin deficient mutant revealed a proliferative profile in contrast to the wildtype USA300 strain that was distinctly cytotoxic. As a vast majority (29/33) of the cytotoxic supernatants contained ≥225 ng/ml α-toxin, whereas all proliferative supernatants had levels below 221 ng/ml (in the 1∶50 dilutions), we speculate that there might be a critical α-toxin concentration determining cytotoxic responses in PBMC. This underscores the value of quantifying the levels of toxins produced and secreted by the strains as this seems to dramatically influence the cellular responses and potentially virulence.

Similarly, a significant association between response profiles, α-toxin levels and the *agr* type of the strain was noted. Significantly increased α-toxin levels were found in the cytotoxic *agr*I and IV types, as compared to the proliferative *agr*II and III strains. The complexity of agr regulation of *S. aureus* virulence factors was emphasized in the report by Geisinger et al [19] in which protein A, α-toxin, PVL and TSST-1 were tested in congenic strains each harboring a unique *agr* allele (I–IV). They found significant variation in the kinetics and degree of the *agr* signal resulting in differential induction of specific virulence factors. Of special interest, is their finding that *agr* I and IV are the earliest and strongest, followed by *agr* II and III, which is the same hierarchical order as we report here in regards to cytotoxicity. The molecular basis for the noted association between toxin production and *agr* types translating into distinct response profiles has yet to be determined but could possibly be linked to varying levels of secreted auto inducing peptides (AIP). Another possibility is that the AIP-AgrC interaction kinetics may vary between the proliferative and cytotoxic isolates, as previous studies [15], [17], [18], [40] have shown that *agr* I and IV AIPs share almost identical primary sequence with only one amino acid difference whereas agr II and III AIPs show greater variations in their primary sequence.

Another interesting aspect is the potential link between genetic background, *agr* type and disease manifestation [18], [41]. Although the link is far from exclusive, *agr* IV are often emphasized as a disease-related isolate [42], [43]. Furthermore Jarraud et al [41] reported a predominance of *agr* II and III in TSST-1 mediated TSS and scarlet fever (90–94%, respectively). Here we report distinct phenotypic response profiles elicited by clinical CA *S. aureus* isolates, which are determined by the combined action of α-toxin and superantigens, and the data shows a striking association to specific *agr* types, such as the *agr* II and III that demonstrated a superantigen-mediated proliferative profile whereas *agr* I and IV were cytotoxic. Whether these phenotypic response profiles reflect specific pathotypes that contribute to distinct disease manifestations can at present time only be speculated upon and can only be addressed by the use of large well-defined clinical cohorts. For instance, the course of bacteremia, toxic shock syndrome and/or necrotizing infections of the skin and lung might be connected to a specific ratio of superantigenic activity versus cytotoxicity, which would determine the overall biological effect on patients’ immune cells. However, we fully appreciate the complexity of identification of pathotypes dictated by toxin-mediated cellular responses. Clinical manifestations and outcome will depend on a variety of factors including among others varying host susceptibility at both the cellular and genetic level as well as site of infection. In addition, patients can present with both necrotizing infections and severe sepsis/septic shock, which raises the currently unexplored question of expression levels and effects of superantigens and cytotoxins systemically and locally. Nevertheless, identification of specific pathotypes would be of great clinical value as it enables recognition of risk patients and allows for epidemiologic surveillance.

Although clinical data are limited, IVIG has been proposed as adjunctive therapy in fulminant invasive *S. aureus* infections and the mechanistic action suggested to involve neutralizing antibodies against superantigens [33], [35], PVL [37] and α-toxin [34]. Here we found that addition of IVIG to PBMC cultures efficiently inhibited both α-toxin-mediated cytotoxicity as well as superantigen-mediated proliferation at physiological concentrations. Thus, our data support a beneficial role of IVIG that through its broad spectrum of antibodies can prevent toxin-mediated damage, including both excessive proliferation as well as cytolytic events.
